# Pseudohypopyon in a Child With Elevated Intraocular Pressure and Progressive Deterioration of Visual Acuity

**DOI:** 10.7759/cureus.97958

**Published:** 2025-11-27

**Authors:** Mierzhati Miershali, Gurnoor S Gill, Mutalifu Obulkasim, Harnaina Bains, Harshal A Sanghvi

**Affiliations:** 1 Department of Ophthalmology, First Affiliated Hospital of Xinjiang Medical University, Ürümqi, CHN; 2 Department of Medicine, Florida Atlantic University Charles E. Schmidt College of Medicine, Boca Raton, USA; 3 Department of Ophthalmology, Broward Health, Pompano Beach, USA; 4 Department of Clinical Trials, Advanced Research, Deerfield Beach, USA; 5 Department of Information Technology and Operations Management, College of Business, Florida Atlantic University, Boca Raton, USA

**Keywords:** elevated intraocular pressure, ophthalmology, pediatric ophthalmology, pseudohypopyon, visual acuity

## Abstract

This case report aims to investigate the etiology of a pseudo-hypopyon in a 10-year-old boy with elevated intraocular pressure and progressive deterioration of visual acuity. The report seeks to elucidate potential underlying causes, clinical features, and diagnostic challenges associated with this rare ocular condition. The patient initially presented with a floating, white, circular object in the left eye, which progressed to redness, pain, and high intraocular pressure despite treatment with anti-inflammatory eye drops. Examination findings included gray-white deposits in the anterior chamber, increased intraocular pressure, and vitreous opacity. The patient’s condition worsened over time, with increased intraocular pressure and decreased visual acuity, leading to consideration of potential diagnoses such as ciliary body medulloepithelioma, pigmented cysticercosis, or retinoblastoma. However, a definitive diagnosis could not be established due to the inability to perform a biopsy or surgical intervention without risking further complications.

## Introduction

A pseudo-hypopyon refers to the presence of anterior chamber material that resembles a true hypopyon but differs in both composition and underlying etiology [1]. While a true hypopyon is composed of leukocytes accumulating in the anterior chamber as a result of infectious or inflammatory processes, a pseudo-hypopyon may consist of neoplastic cells, lipid or crystalline deposits, fungal elements, lens-derived material, or aggregated inflammatory debris. Distinguishing between these entities is essential because their causes, clinical implications, and management strategies vary significantly [1].

Although pseudo-hypopyon is already a rare finding in routine ophthalmic practice, its occurrence in pediatric patients is even more uncommon [2]. Reported etiologies in children include intraocular tumors such as retinoblastoma or medulloepithelioma, infectious processes such as fungal endophthalmitis or ocular cysticercosis, and chronic or recurrent inflammatory conditions [1]. The rarity of pediatric pseudo-hypopyon contributes to diagnostic uncertainty, especially when clinical features deviate from classical descriptions [2,3].

Traditionally, pseudo-hypopyon is described as forming a fluid level that shifts with changes in head position. However, this is not a universal characteristic [2]. Depending on the underlying cause, pseudo-hypopyon may present as discrete spherical aggregates, particulate deposits, or non-layering material within the anterior chamber [2]. These atypical morphologies can obscure the diagnosis, especially when they do not exhibit the expected dependent layering or sedimentation seen in inflammatory hypopyons [1]. Recognizing such variations is important to prevent misinterpretation and to inform an appropriate diagnostic evaluation [3].

Given these challenges, a structured approach to evaluation, including slit-lamp examination, gonioscopy, ultrasonography, and multimodal imaging, is essential to differentiate among neoplastic, infectious, and inflammatory causes [2]. Early and accurate identification of pseudo-hypopyon is particularly critical in children, as delays in diagnosis may increase the risk of irreversible ocular damage, including elevated intraocular pressure, optic nerve compromise, and progressive visual decline [3].

This case highlights an atypical pediatric presentation featuring non-layering spherical deposits in the anterior chamber, underscoring the importance of recognizing morphological variability in pseudo-hypopyon and the need for thorough diagnostic investigation when classical features are absent [2].

## Case presentation

Prior history

We present a 10-year-old boy who presented to the clinic with a left eye glare accompanied by a visual obstruction, which began five months ago without any apparent cause. During the first visit, the patient's mother accidentally discovered a floating, white, circular object in the left eye. In the second visit, the patient experienced redness and pain in the left eye, along with visual obstruction, and therefore visited a local hospital for treatment. The local hospital administered a treatment of anti-inflammatory eyedrops (details unspecified). Following this visit, the child's sensation of visual obstruction intensified. In light of these developments, the decision was made to seek further evaluation and treatment at a higher-level hospital to address the worsening visual obstruction. This hospital found that the child had high intraocular pressure (IOP) and diagnosed left eye uveitis and secondary glaucoma. The prescribed treatment regimen is included in Table 1.

**Table 1:** Patient Medication Regimen

Medication	Dosage Frequency	Eye
Tobramycin/Dexamethasone (Eye Drops)	QID	OS
Tobramycin/Dexamethasone (Eye Ointment)	QD	OS
Pranoprofen (Eye Drops)	QID	OS
Timolol (Eye Drops)	BID	OS
Brimonidine (Eye Drops)	BID	OS
Compound Tropicamide (Eye Drops)	TID	OS
Tafluprost (Eye Drops)	TID	OS

After discharge from the hospital, the patient continued to use the prescribed eye drops. Although the patient continued the full anti-glaucoma regimen - timolol, brimonidine, and tafluprost - the IOP remained markedly elevated. This inadequate response is likely because the secondary glaucoma resulted from mechanical obstruction of aqueous outflow by the progressive accumulation of white spherical deposits, inflammatory debris, and exudative material in the anterior chamber, as documented on slit-lamp examination and B-scan imaging. In this setting, topical medications alone are often unable to overcome compromised trabecular outflow, resulting in persistently high IOP.

**Figure 1:**
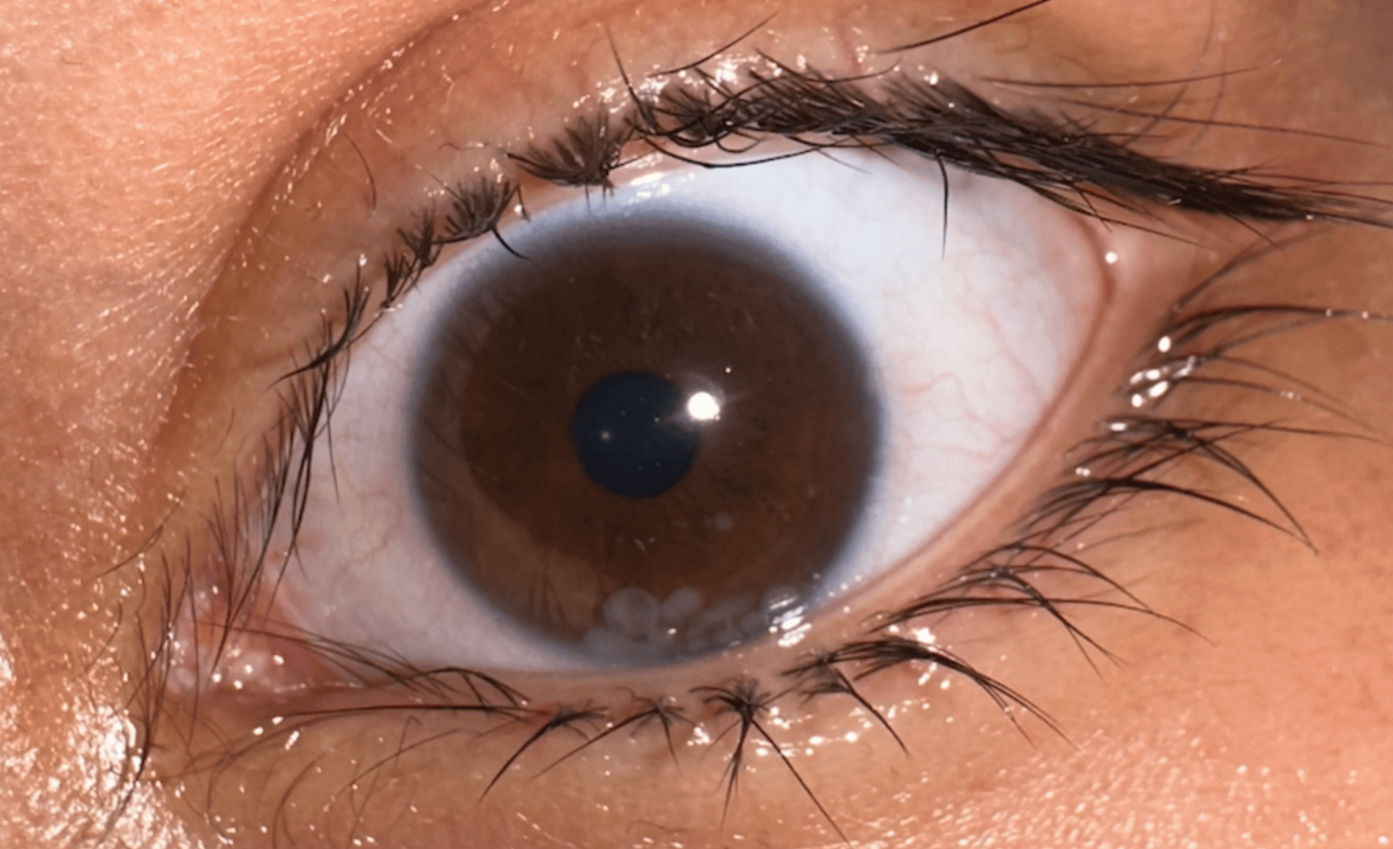
Visual Depiction of Spherical Foreign Bodies in Patient's Eye The spherical foreign body inside the eye moves with gravity.

The number of white spherical floaters in the anterior chamber increased, as shown in Figure 1. After repeated visits to the hospital, they were advised to seek further evaluation and management at the First Affiliated Hospital of Xinjiang Medical University. Then, the patient visited our outpatient department for the third time; after a thorough examination, the patient was admitted to our ophthalmology inpatient department for further evaluation.

**Figure 2:**
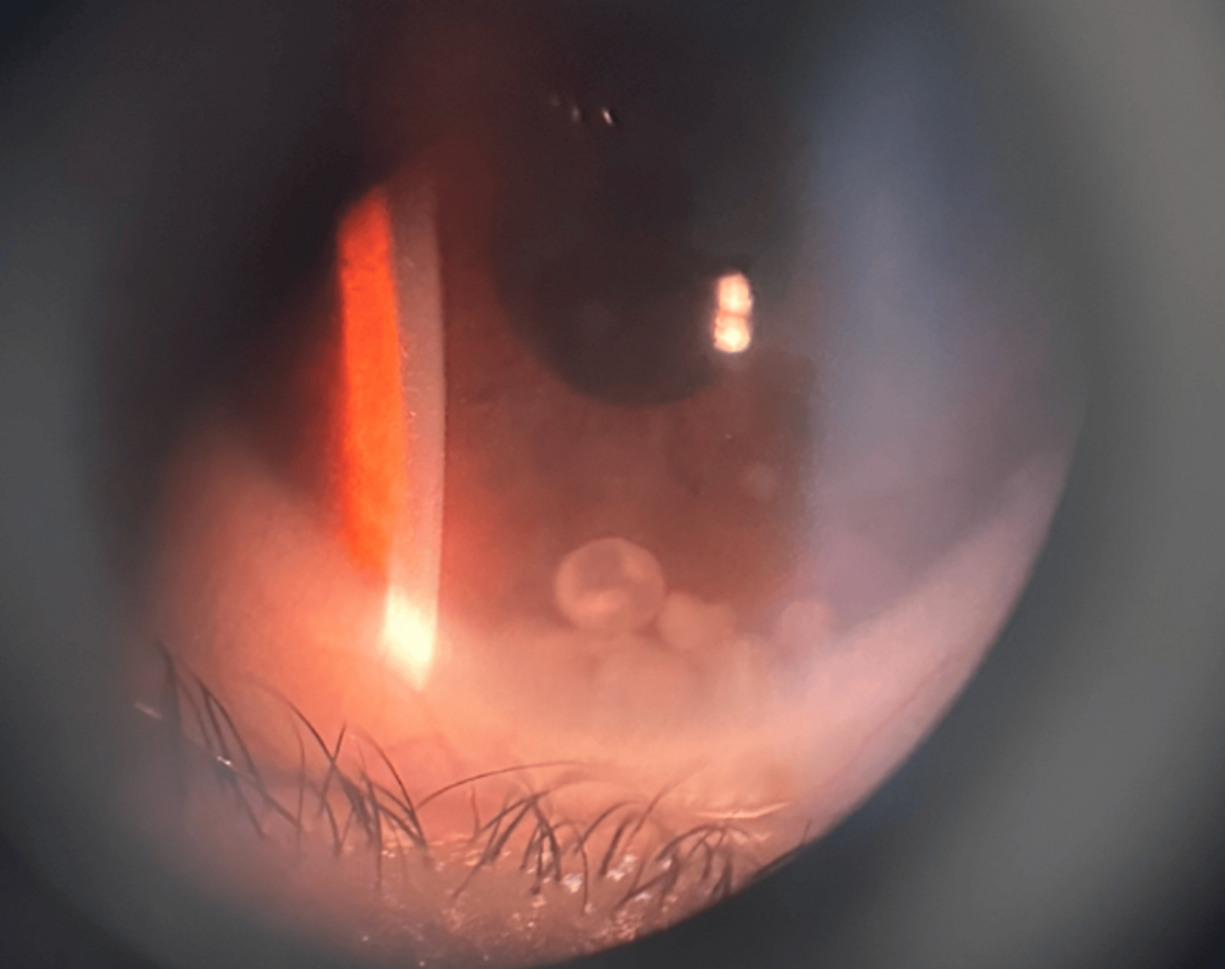
Slit Lamp Exam Findings

Physical examination revealed visual acuity of 1.0 in both eyes. IOP, measured by non-contact tonometry, was elevated in the left eye at 48 mmHg and standard in the right at 14 mmHg. A slit-lamp examination of the left eye showed conjunctival congestion and lamb fat-like keratic precipitates on the cornea, as shown in Figure 2.

**Figure 3:**
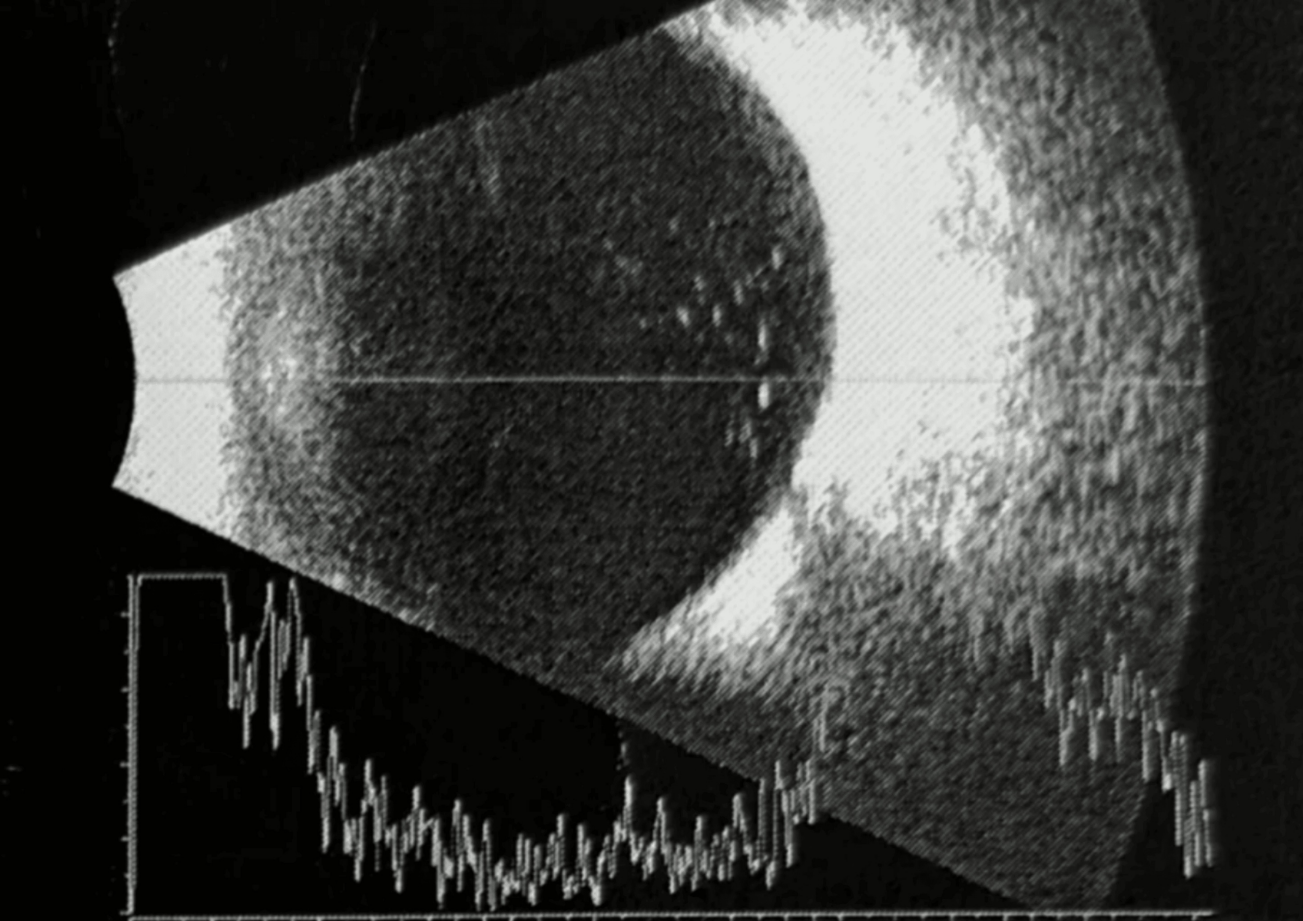
B-scan Ultrasonography of the Globe Demonstrating Preserved Posterior Segment Anatomy

The anterior chamber had a central depth of approximately 3.40 mm with standard peripheral depth, containing bubble-like floating granules, fine punctate low-echo spots, multiple medium-echo clumps, and vesicular structures, accompanied by a significant aqueous flare (+++). At the 3 and 9 o'clock positions, linear medium-echo ciliary zonules were visible and intact. Vitreous examination of the left eye revealed opacity with localized nodular exudative changes consistent in sequential B-scans taken one day apart, as shown in Figure 3.

**Figure 4:**
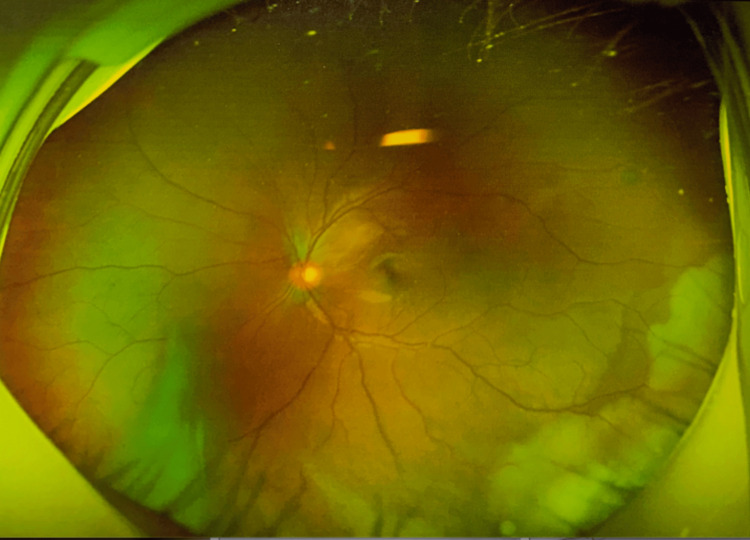
Indirect Physical Exam Findings

Fundus examination by indirect ophthalmoscopy and scanning laser ophthalmoscopy demonstrated clear optic disc margins with standard color and a cup-to-disc ratio of approximately 0.3, as shown in Figure 4. The central foveal reflex was absent, and no visible retinal hemorrhages or exudates. Subretinal exudative changes were noted inferior temporally.

**Figure 5:**
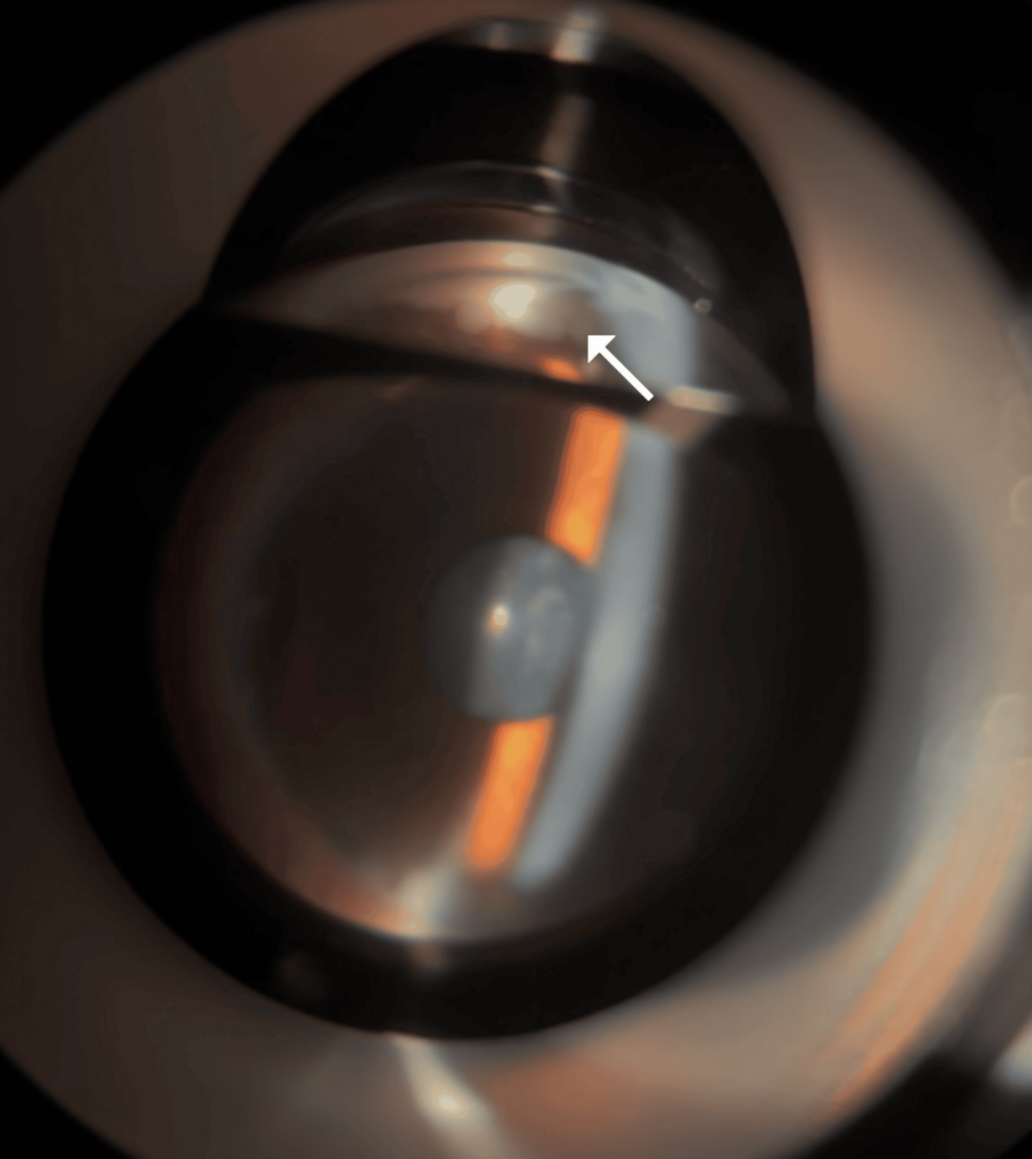
Gonioscopic View of the Anterior Chamber Angle The arrow indicates the gray-white spherical lesion located at the ciliary body root.

Gonioscopy and slit-lamp examination identified a spherical mass attached to the ciliary body root, as shown in Figure 5. Examination of the right eye showed no abnormalities, with visual acuity of 1.0 and IOP of 14 mmHg.

Blood biochemistry, cranial CT, and immunological tests revealed no abnormalities. Examination showed spherical intraocular foreign bodies that moved with gravity. Concerned about potential tumor rupture and dissemination during biopsy, we recommended discharge and referral to Eye and ENT Hospital of Fudan University, Shanghai, due to risks associated with uncertain intraocular foreign-body removal.

Follow-up at nine months

The patient’s visit to Shanghai was delayed due to financial reasons. Therefore, the patient returned to our outpatient department for a reassessment. On examination, the patient's condition had deteriorated, with visual acuity now 1.0 OD and 0.4 OS, and IOP 18 mmHg OD and 45 mmHg OS.

**Figure 6:**
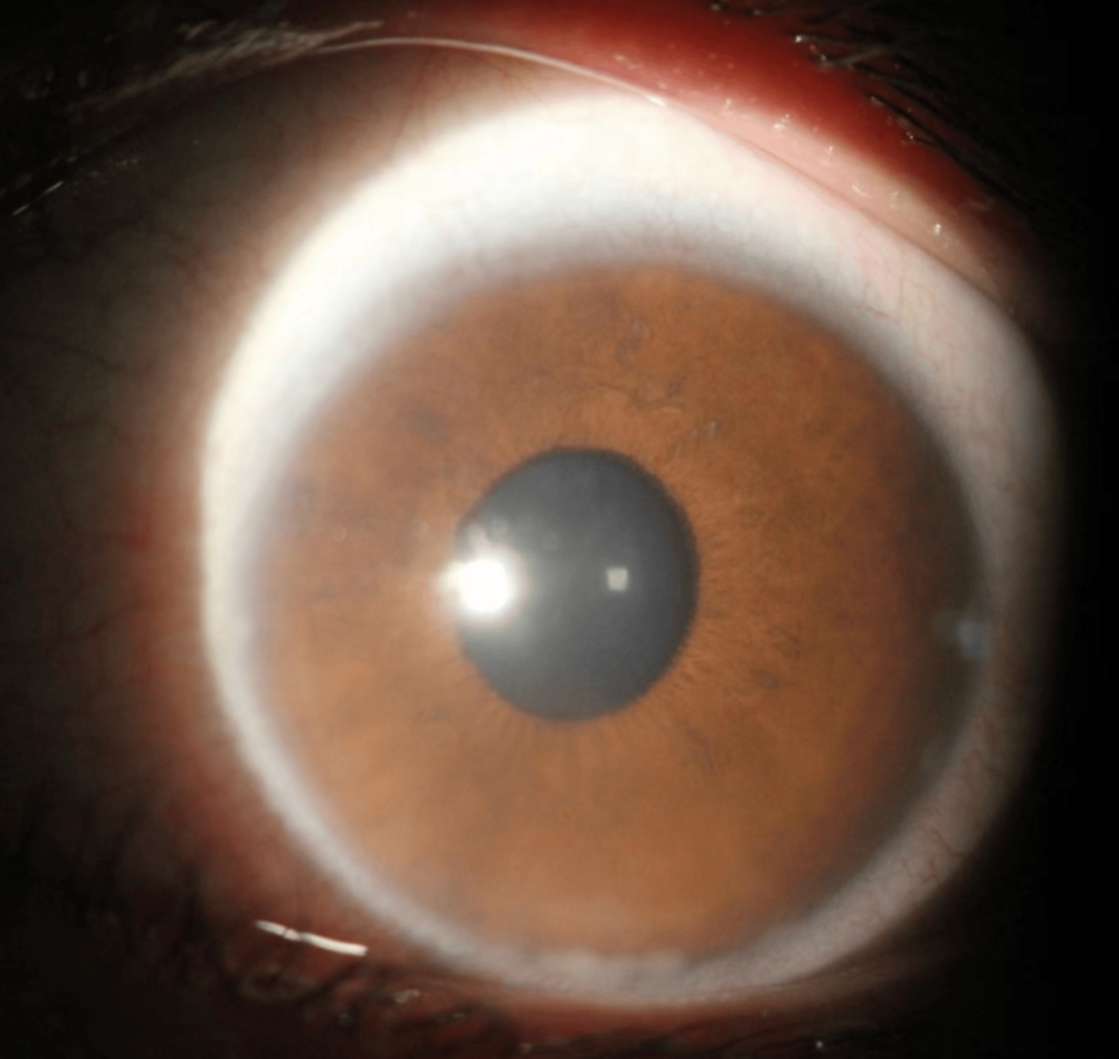
Follow-Up Depiction of Foreign Body

The left eye showed conjunctival congestion and corneal edema. The anterior chamber depth was approximately 3 mm centrally, with peripheral depths ranging from 1/2 to 1 mm. Bubble-like floating granules were observed in the anterior chamber, and the anterior chamber exhibited a flare graded as ++, as shown in Figure 6. The iris texture was clear, and the pupil was round and light-reactive. The lens was clear, but a vitreous haze was present. The optic disc appeared pale, with a cup-to-disc ratio of approximately 0.9. The foveal reflex was not visible, and the retina was in position.

Final comments

The patient, scheduled for surgical intervention, had to cancel the procedure unexpectedly due to a serious illness in his father the day before the operation. During follow-up communication, additional information was obtained regarding the status of the intraocular floating material previously observed in the anterior chamber. According to the patient, the floating material had spontaneously disappeared before the preoperative examination, a finding confirmed by ophthalmological evaluation of the anterior segment upon admission for planned surgical intervention. Due to the severity of the cataract, posterior segment evaluation was impossible.

The patient underwent phacoemulsification of the left eye, posterior capsulotomy, anterior vitrectomy, and goniosynechialysis to address the cataract, manage potential complications from the previously observed anterior chamber material, and optimize IOP control. The surgery was performed at the First People's Hospital in Kashgar.

Importantly, earlier in the disease course, before the cataract had fully matured, posterior segment visualization was still possible. Fundus examination at that time demonstrated clear optic disc margins and inferior temporal subretinal exudative changes, indicating that sufficient optical clarity remained during the initial evaluations. Over the following months, however, the patient experienced progressive lens changes and persistent elevation of IOP. By the time of preoperative assessment, the cataract had advanced substantially, preventing posterior segment visualization and even precluding axial length measurement with the IOLMaster. This progression explains why the fundus was initially visible but later became obscured by the dense cataract.

Preoperative biometry showed an axial length of 23.91 mm in the right eye, while the axial length of the left eye could not be measured using optical biometry due to the advanced cataract; A-scan ultrasound measurement of the left eye revealed an axial length of 24.88 mm. Anterior chamber depth was 3.71 mm in the right eye and 2.43 mm in the left, consistent with a shallow anterior chamber in the affected eye. Keratometry demonstrated curvatures of 41.90 D at 180° and 41.67 D at 90° in the right eye, and 41.40 D at 216° and 41.80 D at 110° in the left eye. The left eye also exhibited a markedly increased lens thickness of 4.78 mm, consistent with the now-advanced cataract. Preoperative corneal endothelial parameters were within normal limits

## Discussion

This case of a 10-year-old boy presenting with a pseudo-hypopyon, elevated IOP, and progressive visual deterioration highlights the complexities of diagnosing and managing rare pediatric ocular conditions, particularly when invasive procedures pose significant risks. Given the diagnostic uncertainty, this report underscores the need for novel, minimally invasive diagnostic techniques and early intervention strategies to optimize patient outcomes.

Posner-Schlossman syndrome (PSS) 

PSS, or glaucomatocyclitic crisis, is a rare ocular condition characterized by recurrent episodes of markedly elevated IOP and mild anterior chamber inflammation [4]. Typically, PSS affects individuals between the ages of 20 and 50, with pediatric cases being uncommon [4]. Although PSS predominantly affects adults, there are rare reports of pediatric cases. For instance, a 13-year-old boy presented with decreased vision, photophobia, halos, and pain in the right eye and was diagnosed with PSS [5]. 

The anterior chamber mass observed in this case may represent an inflammatory aggregation or amalgamation of inflammatory cells, fibrin, and proteinaceous material rather than a discrete, solid mass [4]. In uveitic and inflammatory ocular conditions, pseudo-hypopyon formations can arise due to cellular accumulation within the anterior chamber, mimicking a tumor-like structure [4]. These findings resolve spontaneously or with treatment as the inflammatory process subsides [5]. Mild anterior chamber reactions, characterized by aqueous flare and pigment dispersion, have been documented in PSS, with these abnormalities resolving between episodes [6-8].

Ocular cysticercosis

Taeniasis occurs when humans ingest undercooked or raw pork contaminated with cysticerci, the larval stage of the tapeworm [9]. Once ingested, the cysticerci develop into adult tapeworms in the intestines, which may cause mild gastrointestinal symptoms or be asymptomatic [9]. Humans can also develop cysticercosis if they ingest T. solium eggs, which hatch and release oncospheres that penetrate the intestinal wall and migrate through the bloodstream to various tissues, including the brain, muscles, and eyes [9]

A similar case study by Swastika et al. presents a nine-year-old Balinese girl with ocular cysticercosis caused by the *Taenia solium* Asian genotype [10]. She exhibited similar symptoms in this study, including redness, tearing, pain in her left eye, and a reduced visual acuity of 6/10 in the affected eye [10]. Slit-lamp examination showed a fibrinous larva-like form in the anterior chamber at approximately 5 × 3 mm [11]. Histopathological examination revealed an immaturecysticercus without hooklets, and mitochondrial DNA analysis confirmed it as *Taenia solium* Asian genotype [10]. Despite negative serology results, the patient underwent successful treatment with normal eye function at a nine-month follow-up [10]. 

Fungal endophthalmitis

Fungal endophthalmitis, most commonly caused by Candida species, can produce a pseudohypopyon consisting of clumped inflammatory debris, fungal elements, and particulate aggregates within the anterior chamber [11]. Unlike bacterial hypopyon, fungal pseudohypopyon may appear as multiple white or cream-colored granules that do not always form a smooth fluid level, instead collecting as irregular deposits that shift or settle variably with gravity [11]. In pediatric patients, fungal endophthalmitis is rare but may result from hematogenous spread, trauma, or postoperative infection [11]. Although our patient’s anterior chamber material exhibited spherical morphology rather than layered sedimentation, fungal etiologies remain an important diagnostic consideration, particularly in cases with chronic inflammation, granularity, or atypical particulate patterns [11].

Lipid or crystalline pseudohypopyon

Another important differential is a lipid or crystalline pseudohypopyon, which may occur in metabolic disorders such as lecithin-cholesterol acyltransferase (LCAT) deficiency, longstanding uveitis, or degenerative ocular conditions [1]. These pseudohypopyons are characterized by refractile, shimmering, or particulate deposits that can appear as discrete floating spheres rather than a continuous fluid layer. Crystalline pseudohypopyons often shift with positional changes but may not produce a uniform fluid level, instead demonstrating clumped or bead-like aggregates similar to those observed in our patient [1]. While metabolic causes were not supported by this patient’s systemic evaluation, the morphology of multiple spherical particles in the anterior chamber warrants consideration of crystalline or lipid-based etiologies within the differential diagnosis [1].

Delayed follow-up and the need for novel diagnostics

Delayed referral due to financial causes played a pivotal role in disease progression, contributing to irreversible optic nerve atrophy and vision loss. Prolonged intraocular hypertension in pediatric patients is known to accelerate retinal ganglion cell degeneration, making early intervention critical [12]. Limited access to tertiary care delayed definitive diagnostic procedures, such as anterior chamber paracentesis, which could have provided earlier insights into the pseudo-hypopyon’s etiology [13]. Financial constraints, geographic barriers, and healthcare system inefficiencies remain critical obstacles to early detection and management in ophthalmology [14]. Addressing these systemic issues through streamlined referral pathways and improved accessibility to subspecialty care is essential to prevent similar cases of vision loss due to delayed intervention.

Aqueous humor analysis using multiplex polymerase chain reaction (PCR) can detect infectious or inflammatory agents with high specificity, reducing the need for invasive sampling [15]. Liquid biopsy techniques examining circulating tumor DNA (ctDNA) or inflammatory cytokines in aqueous humor hold promise for distinguishing malignant from benign conditions [16]. Artificial intelligence (AI)-assisted imaging analysis could enhance early pattern recognition, improving clinical decision-making [17]. Integrating these technologies into routine pediatric ophthalmology practice could lead to earlier diagnosis and better patient outcomes.

Limitations

The spontaneous resolution of the intraocular material before the planned surgical intervention significantly limited the ability to perform a biopsy and establish a definitive diagnosis, leading to a lack of histopathological confirmation and reliance on differential diagnosis. This restriction hinders the precise classification of the pseudo-hypopyon’s etiology, contributing to diagnostic uncertainty. While serial imaging was conducted, the absence of advanced diagnostic modalities such as anterior segment optical coherence tomography (AS-OCT) or ultrasound biomicroscopy (UBM) limited the structural characterization of the anterior chamber mass, which could have provided additional diagnostic clarity. Furthermore, delayed referrals due to financial and logistical constraints may have exacerbated disease progression, thereby reducing the accuracy of early-stage pathology assessments. The absence of longitudinal follow-up and genetic or molecular testing further limits the ability to determine potential underlying hereditary or systemic associations. Future cases would benefit from earlier intervention, comprehensive imaging, and molecular diagnostics to improve accuracy and optimize patient outcomes. These studies underscore the need for integrating these innovative techniques into clinical practice to improve patient outcomes.

## Conclusions

The case of a 10-year-old boy presenting with pseudo-hypopyon exemplifies the diagnostic difficulties faced in ophthalmology, mainly when invasive procedures carry substantial risks. A conclusive diagnosis remains elusive despite comprehensive evaluations and differential diagnoses, including ciliary body medulloepithelioma, ocular cysticercosis, and retinoblastoma. This challenge primarily stems from the constraints on surgical or histological interventions that could compromise the patient's eye integrity. Consequently, this scenario underscores the urgent need for innovative diagnostic methods and the application of astute clinical judgment in addressing rare and complex ocular conditions. In this case, the patient's vision in the left eye has deteriorated significantly over the past year. IOP remains high despite the use of medication, and optic nerve atrophy has occurred. This progression underscores the crucial importance of accurate diagnosis and effective management in complex situations.
